# The usefulness of pleural fluid presepsin, C-reactive protein, and procalcitonin in distinguishing different causes of pleural effusions

**DOI:** 10.1186/s12890-018-0740-3

**Published:** 2018-11-23

**Authors:** Naoki Watanabe, Tomoya Ishii, Nobuyuki Kita, Nobuhiro Kanaji, Hiroyuki Nakamura, Nobuki Nanki, Yutaka Ueda, Yasunori Tojo, Norimitsu Kadowaki, Shuji Bandoh

**Affiliations:** 1 Department of Internal Medicine, Hematology, Rheumatology and Respiratory Medicine, Faculty of Medicine, Kagawa University, 1750-1 Ikenobe, Miki-cho, Kita-gun, Kagawa 761-0793 Japan; 2 Department of Respiratory Medicine, Sakaide City Hospital, 3-1-2, Kotobuki-cho, Sakaide, Kagawa 762-8550 Japan; 3 Department of Respiratory Medicine, Sanuki Municipal Hospital, 387-1, Ishidahigashi-kou, Sangawa-cho, Sanuki, Kagawa 769-2393 Japan; 4 Department of Respiratory Medicine, Kagawa Prefectural Central Hospital, 1-2-1, Asahimachi, Takamatsu, Kagawa 760-8557 Japan; 5 Department of Respiratory Medicine, National Hospital Organization Takamatsu Medical Center, 8, Shindencho-otsu, Takamatsu, Kagawa 761-0193 Japan

**Keywords:** Presepsin, C-reactive protein, Procalcitonin, Empyema, Pleural effusion

## Abstract

**Background:**

We aimed to determine the presepsin concentration in pleural fluid from patients with pleural effusions of different aetiologies and to compare its diagnostic value with that of pleural fluid C-reactive protein (CRP) and procalcitonin (PCT).

**Methods:**

We enrolled 132 patients with pleural effusion who underwent diagnostic evaluation, and we classified them into six categories: empyema, parapneumonic effusion, tuberculous effusion, malignant effusion, paramalignant effusion, and transudate effusion. Additionally, all pleural effusions were categorised as infectious or non-infectious effusions.

**Results:**

Receiver operating characteristic analysis was used to evaluate diagnostic performance. When diagnosing empyema, the marker with the highest sensitivity was pleural fluid presepsin (cut-off: 754 pg/mL; sensitivity: 90.9%, specificity: 74.4%) and that with the highest specificity was pleural fluid CRP (cut-off: 4.91 mg/dL; sensitivity: 63.6%, specificity: 89.3%). Pleural fluid PCT tended to be lower in patients with empyema than in those with parapneumonic effusion, but this was not useful for the diagnosis of empyema. When diagnosing infectious pleural effusion, a combination of pleural fluid CRP (cut-off: 2.59 mg/dL) and presepsin (cut-off: 680 pg/mL) produced the highest diagnostic accuracy (83.3%).

**Conclusions:**

Pleural fluid presepsin was found at high levels in patients with empyema and parapneumonic effusion. This pattern closely resembles the previously reported pattern of pleural fluid CRP. Some combinations of pleural fluid inflammatory markers may be more clinically useful than these markers in isolation.

## Background

To investigate the aetiology of pleural effusion, a variety of examinations are typically performed, including pleural fluid cell and differential white blood cell counts, cytological examination, and bacterial culture following Light’s criteria [1]. However, the information obtained by these methods is limited, and delays in the diagnosis and the initiation of appropriate therapy for infectious effusions can increase the rate of complications. Therefore, rapid diagnosis and determination of whether the cause of pleural effusion is infection are beneficial in the treatment of this condition.

Although clinical microbiology testing can confirm the presence of infection, a positive culture is observed in only about 60% of parapneumonic effusions, and the time required to obtain a positive culture can be prolonged [2]. Various pleural biomarkers have therefore been investigated as methods for differentiating infectious pleural effusion [3–5].

Presepsin, also known as soluble CD14 subtype, is a protein reported to be increased specifically in the blood of patients with sepsis [6] and is therefore used to predict sepsis in patients in emergency or intensive care units [7]. Following stimulation by pathogens, presepsin is released via shedding from the surface of various types of immune cells, including macrophages, monocytes, and neutrophils, which are implicated in phagocytosis and the cleavage of membrane CD14 by lysosomal enzymes from granulocytes in response to bacterial infection [8]. Several studies have confirmed that presepsin is a more specific and sensitive marker for the diagnosis of sepsis compared with C-reactive protein (CRP), interleukin-6 (IL-6), or procalcitonin (PCT) [7], [9–11]. However, few studies have investigated the diagnostic value of presepsin levels in body fluids.

The aim of the present study was to evaluate the pleural fluid concentrations of presepsin, CRP, and PCT in patients with pleural effusions of various causes and to explore the usefulness of these markers in predicting an infectious aetiology.

## Methods

### Study design

In this cross-sectional study, we set the target number of cases based on feasibility. An average of 30 thoracentesis procedures are performed at the participating institutions annually. Allowing for ineligible patients and a consent rate of 90%, the target number of cases was set at 135. This study protocol was approved by the institutional review boards of Kagawa University Hospital and all other participating institutions. All patients provided written informed consent prior to participation.

Between November 2015 and March 2017, 145 patients aged ≥18 years with pleural effusion and planned thoracentesis for diagnosis were initially enrolled in the study. Among these, seven patients with chronic maintenance dialysis or continuous hemodiafiltration and six patients with severe renal failure (GFR of < 15 mL/min/1.73 m^2^) were excluded from further participation.

### Diagnostic criteria

Pleural fluid samples were collected from 132 patients. Each diagnosis was made according to the diagnostic criteria described below. The cases were also categorised as infectious or non-infectious effusions.

We classified the aetiology of pleural effusion into seven categories. 1) Empyema was defined as a grossly purulent pleural effusion accompanied by bacteria detected by Gram staining or a positive culture for bacteria. 2) Parapneumonic effusion was defined as pleural effusion associated with bacterial pneumonia. 3) Tuberculous pleural effusion was based on the presence of a caseous granuloma in the pleural biopsy and/or a positive culture for *Mycobacterium tuberculosis* in the pleural fluid or biopsy material or a positive sputum culture with an exudative pleural effusion and both a clinical and radiological response to anti-tuberculous treatment. 4) Malignant effusion was diagnosed when malignant cells were found in the pleural fluid or in a biopsy specimen. 5) Paramalignant effusion was diagnosed in patients with a known malignancy or subsequent diagnosis of malignancy but with negative cytology and no obvious alternative diagnosis. 6) Transudate effusion was diagnosed by the attending physician based on Light’s criteria as well as the general condition of the patient. 7) Any pleural effusion not meeting one of the above diagnostic criteria was categorised as an unclassifiable pleural effusion.

Notably, the cases of unclassifiable pleural effusion were divided into infectious or non-infectious effusions, based on the judgment of the attending physician and the reactivity of the condition to antimicrobial drugs. Therefore, the infectious pleural effusion cases in this study included all the patients with empyema, parapneumonic effusion, and tuberculous pleural effusion as well as those with unclassifiable pleural effusion who were clinically diagnosed as having infectious pleural effusion.

### Procedures

Thoracentesis was performed under local anaesthesia. Samples of pleural fluid were immediately subjected to routine examinations for analysis (e.g. pH, total protein, glucose, and lactate dehydrogenase), total and differential cell counts, and cytological and microbiological examination. Pleural fluid was collected in a serum-separating tube for CRP measurement and in a tube containing EDTA for presepsin and PCT measurement. Samples were centrifuged at 1200×*g* for 5 min at 4 °C, and the resulting supernatants were stored at − 30 °C until they were assayed. Simultaneously, venous blood was obtained and analysed for white blood cell count and for lactate dehydrogenase, total protein, blood urea nitrogen, creatinine, and CRP content in addition to any other appropriate assessments ordered by the attending physician. Aliquots of blood plasma were stored at − 30 °C prior to the assessment of presepsin and PCT levels.

### Biomarker assays

Assays for the three inflammatory markers, presepsin, CRP, and PCT, were performed on the cell-free supernatants of pleural fluid and blood plasma samples. All samples were tested in random order by technicians blinded to the clinical diagnosis.

Pleural fluid CRP measurement was performed by a latex-enhanced immunoturbidimetric assay at an external clinical laboratory testing facility (SRL Inc., Tokyo, Japan). PCT concentrations in pleural fluid and blood plasma were measured using the Wako i30 micro-total analysis system (μTAS) and Wako–BRAHMS PCT assay (Wako Pure Chemical Industries, Ltd., Osaka, Japan), respectively. Presepsin concentrations in pleural fluid and blood plasma were determined using a compact automated immunoanalyser based on a chemiluminescent enzyme immunoassay (PATHFAST; Mitsubishi Chemical Medience Corporation, Tokyo, Japan).

### Statistical analysis

Data are presented as means ± standard deviation (SD) for data with a normal distribution and as medians with interquartile ranges in parentheses for skewed data. Normality of distribution was ascertained using the Shapiro–Wilk test. For comparing categorical data, a chi-squared test was performed. For the evaluation of diagnostic performance, receiver operating characteristic (ROC) analysis was performed, ROC curves were generated by plotting sensitivity against 1-specificity, and the area under the curve (AUC) with 95% confidence intervals (CI) was calculated. The Youden index was used to identify the cut-off values with potential diagnostic significance. All statistical analyses were performed using JMP software version 12.2.0 (SAS institute Inc., Cary, NC, USA), and *p*-values of < 0.05 were considered statistically significant.

## Results

### General characteristics of pleural effusions

Of the 132 patients classified as having pleural effusions, 122 (92.4%) were diagnosed with exudative effusion and 10 (7.6%) were diagnosed with transudate effusion. Additionally, 38 (28.8%) patients were diagnosed with infectious pleural effusions, while 94 (71.2%) were diagnosed with non-infectious pleural effusions.

The exudative effusion group was further divided into the following six subgroups according to the diagnosis: empyema, 11 (9.0%); parapneumonic effusion, 16 (13.1%); tuberculous pleural effusion, 9 (7.4%); malignant effusion, 46 (37.7%); paramalignant effusion, 13 (10.7%); and unclassified effusion, 27 (22.1%). Although the attending physician investigated thoroughly to determine the cause of pleural effusion, the cause remained unknown in 17 cases. Among the other 10 cases of unclassified effusion, there were 2 cases associated with pneumothorax, 2 cases of chronic empyema, 1 case associated with collagen disease, 1 case associated with trauma, 1 case associated with interstitial pneumonia, 1 case associated with asbestos, 1 case of chylous pleural effusion, and 1 case of reactive pleural effusion due to *Clonorchis sinensis*. The chronic empyema cases categorised as unclassified effusions did not meet the diagnostic criteria of empyema for this study. Of the 27 unclassifiable pleural effusion cases, only the two chronic empyema cases were judged as being infectious pleural effusion. The demographic data and the pleural fluid characteristics of the 132 patients included in the present study are shown in Table 1.

**Table 1:** Demographic data and pleural fluid characteristics (*n* = 132)

	Empyema (*n* = 11)	Parapneumonic (*n* = 16)	Tuberculous (*n* = 9)	Malignant (*n* = 46)	Paramalignant (n = 13)	Transudates (*n* = 10)	Unclassified (*n* = 27)
Age (years)	74.3 ± 11.1	76.7 ± 10.1	76.1 ± 17.4	74.5 (67, 82.3)	77.2 ± 10.9	80.7 ± 9.5	79 (73, 88)
Sex (male/female)	9/2	13/3	4/5	25/21	9/4	9/1	20/7
Pleural fluid white blood cell count	6355 (2160, 23,693)	1870 (963, 6338)	931 (782, 1325)	1290 (890, 2355)	1532 ± 999	658 ± 356	1045 (613, 4029)
Pleural fluid lymphocytes (%)	22.3 ± 22.4	33.3 ± 26.5	81.1 ± 11.3	36.5 (17.3, 63.5)	57.3 ± 28.9	54.9 ± 16.1	73 (35.5, 86.5)
Pleural fluid neutrophils (%)	67.7 ± 31.1	45.5 (8.9, 77.1)	5.2 ± 5.2	5.0 (1.4, 20)	10.0 (4.5, 16.5)	16.1 ± 16.1	8.0 (1.1, 15)
Pleural fluid glucose (mg/dL)	10 (1, 107)	160 ± 62	104 ± 28	106 (74, 123)	104 ± 33	117 (110, 141)	93 ± 43
Total protein in pleural fluid (g/L)	3.8 ± 1.1	3.9 ± 0.6	4.2 ± 0.9	4.6 (3.8, 5.1)	3.9 (3.5, 4.8)	2.7 ± 1.0	4.5 ± 1.1
Total protein in serum (g/L)	6.0 ± 1.1	6.3 (5.8, 6.6)	6.5 ± 0.6	6.8 ± 0.7	6.9 ± 0.7	6.0 (5.3, 6.4)	7.1 ± 1.2
Total protein in pleural fluid/serum ratio	0.65 ± 0.19	0.67 (0.54, 0.71)	0.64 ± 0.11	0.67 (0.58, 0.72)	0.62 ± 0.10	0.44 ± 0.14	0.63 ± 0.14
Pleural fluid LDH (U/L)	1076 (474, 2522)	496 ± 247	396 ± 250	410 (267, 775)	234 (164, 392)	117 (94, 128)	232 (171, 543)
Serum LDH (U/L)	189 ± 71	254 (194, 325)	205 (186, 245)	269 (237, 395)	240 ± 68	250 (209, 363)	191 (152, 238)
LDH pleural fluid/serum ratio	6.22 (1.41, 29.67)	1.45 (0.90, 3.41)	1.77 ± 1.02	1.25 (0.97, 2.92)	0.88 (0.76, 1.77)	0.42 ± 0.17	1.24 (0.85, 2.45)

LDH, lactate dehydrogenase; Data are presented as the mean ± SD for normally distributed data or as the median (interquartile ranges) for skewed data

### Pleural fluid levels of presepsin, CRP, and PCT

#### Presepsin measurements

**Fig. 1:**
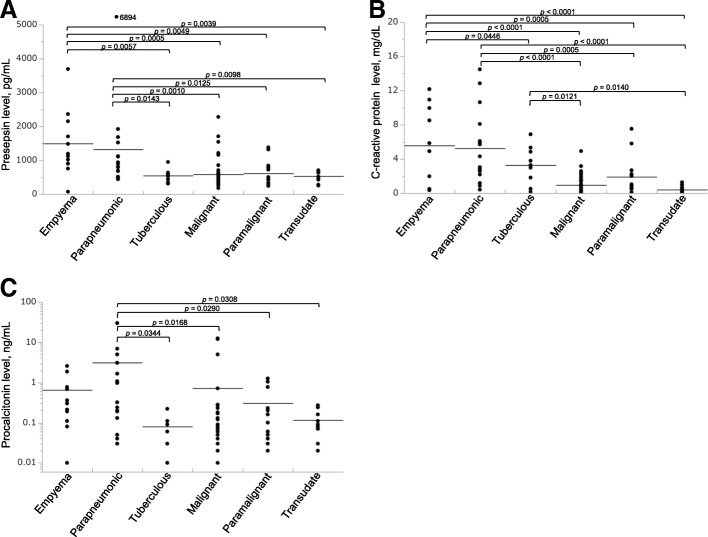
Pleural fluid presepsin, CRP, and PCT levels. (**a–c**) Pleural fluid presepsin (**a**), CRP (**b**), and PCT (**c**) levels in the different diagnostic subgroups. Individual values are plotted. Bars represent the means of the values, and *p-*values are shown between only groups with statistically significant differences

Pleural fluid presepsin levels were significantly higher in patients with empyema or parapneumonic effusion compared with patients who had other types of effusions. However, no difference in the pleural fluid presepsin level was observed between these two types of effusions (Table 2, Fig. 1a). Presepsin levels were significantly higher in pleural fluid than in blood for all types of effusions.

**Table 2:** Levels of presepsin, CRP, and PCT in the pleural fluid and blood (*n* = 105)

	Empyema (*n* = 11)	Parapneumonic (*n* = 16)	Tuberculous (*n* = 9)	Malignant (*n* = 46)	Paramalignant (*n* = 13)	Transudates (*n* = 10)
Presepsin (pg/mL)						
PF	1496 ± 971	854 (682, 1423)	548 ± 188	463 (347, 609)	438 (319, 812)	531 ± 172
Blood	776 ± 362	395 (251, 851)	392 ± 183	258 (162, 318)	259 (200, 322)	402 ± 144
*p*-value	0.0139	0.0330	0.0019	< 0.0001	0.0111	0.0125
CRP (mg/dL)						
PF	5.57 ± 4.40	3.67 (2.28, 7.59)	3.29 ± 2.22	0.55 (0.21, 1.48)	0.90 (0.53, 2.46)	0.41 ± 0.43
Blood	19.11 ± 9.88	8.04 (2.97, 19.35)	8.06 ± 6.18	1.62 (0.49, 5.80)	3.12 (1.37, 10.66)	1.00 (0.25, 2.18)
*p*-value	0.0001	0.0114	0.0047	< 0.0001	0.0069	0.0130
PCT (ng/mL)						
PF	0.30 (0.11, 0.77)	0.28 (0.07, 2.72)	0.08 ± 0.06	0.05 (0.03, 0.10)	0.10 (0.04, 0.50)	0.12 ± 0.08
Blood	1.43 (0.25, 3.26)	0.30 (0.13, 2.13)	0.08 ± 0.05	0.06 (0.03, 0.12)	0.06 (0.04, 0.23)	0.12 ± 0.10
*p*-value	0.0272	ns	ns	ns	ns	ns

Data are presented as the mean ± SD for normally distributed data or median (interquartile ranges) for skewed data. PF: pleural fluid

The diagnostic performance of pleural fluid presepsin values as determined from a ROC analysis is presented in Table 3. Pleural fluid presepsin may represent a useful marker for the differentiation of empyema from other types of effusions. Using a cut-off point of 754 pg/mL, pleural presepsin presented 90.9% sensitivity and 74.4% specificity for the diagnosis of empyema.

**Table 3:** Diagnostic performance of pleural fluid presepsin based on the ROC analysis (*n* = 132)

	Optimal cut-off point (pg/mL)	Sensitivity (%)	Specificity (%)	+LR	−LR	PPV (%)	NPV (%)	AUC	Accuracy (%)
Emp vs. other	≥754	90.9	74.4	1.39	0.12	24.4	98.9	0.809	75.8
Emp and PE vs. other	≥680	85.2	74.3	3.31	0.20	46.0	95.1	0.803	76.5
Emp, PE, and TB vs. other	≥680	66.7	72.9	2.46	0.46	48.0	85.4	0.728	71.2
PE vs. other (excluding Emp)	≥680	81.3	74.3	3.16	0.25	32.5	96.3	0.785	75.2
Infectious vs. non-infectious	≥680	68.4	74.5	2.68	0.42	52.0	85.4	0.746	72.7

*Emp* empyema, *PE* parapneumonic effusions, *TB* tuberculosis, +*LR* positive likelihood ratio, −*LR* negative likelihood ratio *PPV* positive predictive value, *NPV* negative predictive value, *AUC* area under the curve

A similar cut-off point was observed in another pair comparison. In distinguishing parapneumonic effusion from other types of effusions (excluding empyema) with a cut-off point of 680 pg/mL, the sensitivity was 81.3% and the specificity was 74.3%.

#### CRP measurements

Pleural fluid CRP levels were significantly higher in patients with empyema, parapneumonic effusion, and tuberculous effusion compared with patients who had malignant or transudate effusions. There was no difference in pleural fluid CRP levels between empyema and parapneumonic effusion cases. Additionally, no difference in pleural fluid CRP levels was observed between malignant effusion, paramalignant effusion, and transudate effusion cases. CRP levels were significantly lower in pleural fluid than in blood for all types of effusions (Table 2, Fig. 1b).

The CRP diagnostic performance based on a ROC analysis of pleural fluid CRP values is presented in Table 4. Pleural fluid CRP was found to represent a useful marker for the diagnosis of empyema; using a cut-off point of 4.91 mg/dL, pleural fluid CRP presented 63.6% sensitivity and 89.3% specificity for the diagnosis of empyema. In distinguishing between infectious effusions and non-infectious effusions, the sensitivity was 65.8% and the specificity was 90.4% when using a pleural fluid CRP cut-off point of 2.59 mg/dL. Pleural fluid CRP showed the highest accuracy compared with pleural fluid presepsin and PCT.

**Table 4:** Diagnostic performance of pleural fluid CRP based on the ROC analysis (*n* = 132)

	Optimal cut-off point (mg/dL)	Sensitivity (%)	Specificity (%)	+LR	−LR	PPV (%)	NPV (%)	AUC	Accuracy (%)
Emp vs. other	≥4.91	63.6	89.3	5.93	0.41	35.0	96.4	0.757	87.1
Emp and PE vs. other	≥2.59	70.4	85.7	4.92	0.35	55.9	91.8	0.834	82.6
Emp, PE and TB vs. other	≥2.59	69.4	90.6	7.40	0.34	73.5	88.8	0.840	84.9
PE vs. other (excluding Emp)	≥2.18	81.3	81.0	4.27	0.23	39.4	96.6	0.859	81.0
Infectious vs. non-infectious	≥2.59	65.8	90.4	6.87	0.38	73.5	86.7	0.820	83.3

*Emp* empyema, *PE* parapneumonic effusions, *TB* tuberculosis, +*LR* positive likelihood ratio −*LR* negative likelihood ratio, *PPV* positive predictive value, *NPV* negative predictive value, *AUC* area under the curve

#### PCT measurements

Pleural fluid PCT levels were significantly higher in patients with parapneumonic effusion compared with patients who had tuberculous effusion, malignant effusion, paramalignant effusion, or transudate effusion. No difference in pleural fluid PCT levels was observed between empyema and parapneumonic effusion cases. Interestingly, pleural fluid PCT levels tended to be lower in empyema cases than in parapneumonic effusion cases. Pleural fluid PCT levels did not differ from blood PCT levels, except in patients with empyema (Table 2, Fig. 1c).

The PCT diagnostic performance based on a ROC analysis of pleural fluid PCT values is presented in Table 5. This analysis revealed that pleural fluid PCT is unsuitable as a marker for the differentiation of empyema because its AUC was < 0.5. However, pleural fluid PCT may be a useful marker in differentiating parapneumonic effusion from other type of effusions (excluding empyema); when using a cut-off point of 0.11 ng/mL, the sensitivity was 75.0% and the specificity was 79.0%.

**Table 5:** Diagnostic performance of pleural fluid PCT based on the ROC analysis (*n* = 132)

	Optimal cut-off point (ng/mL)	Sensitivity (%)	Specificity (%)	+LR	−LR	PPV (%)	NPV (%)	AUC	Accuracy (%)
Emp vs. other	≤2.57	100.0	5.8	1.06	0.00	8.8	100.0	0.244	13.6
Emp and PE vs. other	≥0.19	70.4	83.8	4.35	0.35	52.8	91.7	0.791	81.1
Emp, PE and TB vs. other	≥0.11	66.7	75.0	2.67	0.44	50.0	85.7	0.718	72.7
PE vs. other (excluding Emp)	≥0.13	75.0	79.0	3.58	0.32	35.3	95.4	0.783	78.5
Infectious vs. non-infectious	≥0.11	63.2	74.5	2.47	0.49	50.0	83.3	0.696	71.2

*Emp* empyema, *PE* parapneumonic effusion, *TB* tuberculosis, +*LR* positive likelihood ratio, −*LR* negative likelihood ratio, *PPV* positive predictive value, *NPV* negative predictive value, *AUC* area under the curve

### The diagnostic accuracy of different marker combinations

The diagnostic accuracy of combined cut-off values for the three inflammatory markers obtained in each ROC analysis is shown in Table 6. To distinguish between empyema and other type of effusions, a pleural fluid presepsin level of > 754 pg/mL and a pleural fluid CRP level of > 4.91 mg/dL yielded the highest accuracy rate (90.9%). Similarly, when distinguishing between infectious pleural effusion and non-infectious pleural effusion, the accuracy rate peaked at 83.3% for pleural fluid presepsin levels of > 680 pg/mL and pleural fluid CRP levels of > 2.59 mg/dL. Furthermore, when distinguishing between parapneumonic pleural effusion and other types of effusions (excluding empyema in both groups), pleural fluid presepsin levels of > 680 pg/mL and pleural fluid CRP levels of > 2.18 mg/dL yielded the highest accuracy rate (90.1%). The combination of pleural fluid PCT levels of < 2.57 ng/mL and pleural fluid CRP levels of > 4.91 mg/dL yielded the second highest accuracy rate (87.9%) in the differentiation of empyema. The combination of PCT and CRP also showed better accuracy than the combination of PCT and presepsin in distinguishing between infectious and non-infectious pleural effusion, parapneumonic pleural effusion, and other types of effusions (excluding empyema in both groups).

**Table 6:** Diagnostic performance of different marker combinations (*n* = 132)

Cut-off value	Sensitivity (%)	Specificity (%)	+LR	−LR	PPV (%)	NPV (%)	Accuracy (%)
Emp vs. other							
Presepsin, 754 pg/mL + CRP, 4.91 mg/dL	63.6	93.4	9.63	0.39	46.7	96.6	90.9
Presepsin, 754 pg/mL + PCT, ≤ 2.57 ng/mL	90.9	78.5	4.23	0.12	27.8	99.0	79.6
CRP, 4.91 mg/dL + PCT, ≤ 2.57 ng/mL	63.6	90.1	6.42	0.40	36.8	96.5	87.9
PE vs. other (excluding Emp)							
Presepsin, 680 pg/mL + CRP, 2.18 mg/dL	68.8	93.3	10.31	0.33	61.1	95.1	90.1
Presepsin, 680 pg/mL + PCT, 0.13 ng/mL	68.8	90.5	7.22	0.35	52.4	95.0	87.6
CRP, 2.18 mg/dL + PCT, 0.13 ng/mL	68.8	92.4	9.02	0.34	57.9	95.1	89.3
Infectious vs. non-infectious							
Presepsin, 680 pg/mL + CRP, 2.59 mg/dL	65.8	90.4	6.87	0.38	73.5	86.7	83.3
Presepsin, 680 pg/mL + PCT, 0.11 ng/mL	55.3	89.4	5.19	0.50	67.7	83.2	79.6
CRP, 2.59 mg/dL + PCT, 0.11 ng/mL	50.0	92.6	6.71	0.54	73.1	82.1	80.3

*Emp* empyema, *PE* parapneumonic effusions, +*LR* positive likelihood ratio, −*LR* negative likelihood ratio, *PPV* positive predictive value, *NPV* negative predictive value

## Discussion

We hypothesised that pleural fluid presepsin, CRP, and/or PCT concentration(s) may be of value in the differentiation between several causes of pleural effusions. Thus, this study investigated the diagnostic performance of these three inflammatory markers in the blood and pleural fluid of a well-characterised population of patients with several types of effusions. We found that pleural fluid presepsin levels were significantly higher in cases of empyema and parapneumonic effusion compared with other types of effusions, as well as in cases with infectious pleural effusions compared with non-infectious effusions, although the pleural fluid presepsin level was not elevated in tuberculous effusion cases. Furthermore, pleural fluid presepsin was found to be the most sensitive of the three tested markers for distinguishing between infectious and non-infectious pleural effusions.

Our findings on pleural fluid presepsin may support a pathophysiological mechanism of the acute phase response during the development of infection. Kiropoulos et al. [12] investigated the levels of CRP, IL-6, and TNF-α in various types of pleural effusions and reported that pleural fluid CRP was likely to reflect the systemic inflammation induced by the local production of IL-6 and TNF-α in the pleural cavity. Notably, although IL-6 and TNF-α levels were higher in the pleural fluid than in the serum, we found that pleural fluid CRP levels were significantly lower than serum CRP levels. Similarly, pleural fluid presepsin levels may reflect local production in the pleural cavity as well as the production mechanism of pleural fluid IL-6 and TNF-α.

Although the biological function of presepsin remains unclear, studies in rabbits [13] showed that its release mechanism is likely to be associated with the phagocytosis and cleavage of microorganisms by lysosomes. This finding supports the high levels of pleural fluid presepsin observed in empyema cases in this study. We suggest that locally increased pleural fluid presepsin levels may be attributed to bacterial phagocytosis in pleural effusion. Furthermore, this connection may be one of the reasons that pleural fluid presepsin does not increase in tuberculous effusion compared with empyema or parapneumonic effusion. As confirmed in the present study (Table 1), the proportion of lymphocytes is relatively high in tuberculous pleural effusion [14]. Thus, there are relatively few macrophages and neutrophils in this condition, and the low numbers of these phagocytes may lead to low presepsin production. Presepsin is produced in the pleural space; therefore, measuring presepsin in the pleural fluid may provide a more accurate marker for distinguishing the cause of pleural effusion than measuring presepsin in the blood.

We found that pleural fluid CRP levels were higher in infectious effusions compared with non-infectious effusions. This result supports the value of pleural fluid CRP measurement in diagnosing infectious effusions. Furthermore, it confirms and extends the findings of previous investigations, in which pleural CRP levels were found to be higher in parapneumonic effusions than in other types of exudates. Izhakian et al. [15] reported that pleural CRP levels were higher in parapneumonic effusion than in other effusion types, with a cut-off value of > 1.38 mg/dL. Pleural CRP had a low positive predicted value (37.6%) but a very high negative predicted value (96.7%), which suggests that it could be a powerful tool for excluding parapneumonic effusion as a diagnosis. Several other studies have investigated the relationship between pleural fluid CRP and the cause of pleural effusions, with similar findings [16, 17]. Although the exact cut-off value varies among studies, pleural fluid CRP levels appear capable of differentiating between infectious effusions and non-infectious effusions. Porcel et al. [4] found that pleural fluid CRP levels of > 10 mg/dL were associated with complicated parapneumonic effusion and with the need for pleural effusion drainage. Here, we examined whether the marker distribution differed between patients treated with antibiotics alone (*n* = 5) and those treated with drainage or surgical treatment (*n* = 11). No difference in PCT or presepsin levels was found between these groups, but CRP levels trended higher in the group with drainage or surgical treatment. Thus, although the number of cases in the present study was small, its results also suggest that pleural fluid CRP levels vary depending on the treatment.

We examined the relationship between pleural fluid PCT levels and different causes of pleural effusions, which has also been investigated by several previous studies. Among those studies, some reported that pleural fluid PCT measurement is useful in differentiating between parapneumonic and other types of effusions, while others have found that it is not useful for this purpose. Wang et al. [18] reported that PCT levels could differentiate empyema and parapneumonic effusion from non-parapneumonic effusion at a cut-off point of 0.18 ng/mL and an AUC of 0.776 (sensitivity, 69.7%; specificity, 72.1%). Lin et al. [19] also reported that PCT could differentiate parapneumonic effusion from non-parapneumonic effusion at a cut-off point of 0.18 ng/mL and an AUC of 0.752 (sensitivity, 66.7%; specificity, 77.4%). However, Porcel et al. [3] determined that PCT levels in pleural fluid were of little value. Our results show that pleural fluid PCT levels tended to be higher in cases of parapneumonic effusion than in other pleural effusions, supporting its potential use as a tool for diagnosing parapneumonic effusion. Although the small number of cases in this study makes it impossible to make definitive conclusions, pleural fluid PCT levels do not necessarily increase in empyema.

A novel finding of our study is that combinations of pleural fluid markers can distinguish pleural fluid from certain causes with a higher accuracy rate than that of any single pleural fluid marker. In the differentiation of empyema, a combination of pleural fluid presepsin and CRP yielded the highest accuracy rate. In clinical practice, simple cut-off values are easier to remember and apply. Notably, we found that changing the cut-off value of presepsin from 754 pg/mL to 750 pg/mL and the CRP cut-off value from 4.91 mg/dL to 4.9 mg/dL, did not affect the diagnostic performance. Although PCT alone was not useful for the differentiation of empyema, it was found that this marker can be a useful tool when combined with CRP or presepsin. However, for distinguishing between infectious effusion and non-infectious effusion, the accuracy rate of combined markers was not improved over that of pleural fluid CRP alone. Further research on the clinical applicability of various marker combinations is required.

This study should be interpreted in the context of certain limitations. First, the sample size was small, and there was a large variation in case accumulation; although multiple study centres participated, patients were enrolled from both respiratory medicine and outpatient wards. This may have limited the power of its findings. Second, diagnosis based on the physician’s judgment may have resulted in the misclassification of some patients, thus compromising the assessment of diagnostic accuracy of the pleural fluid markers. However, as the diagnoses of pleural effusions were performed by board-certified members of the Japanese Respiratory Society at each institution and were made following the same diagnostic criteria, we consider the diagnosis method to have been relatively standardised. Third, dialysis patients and patients with severe renal failure were not included in this study; thus, the findings of this study may not be applicable to patients with severe renal failure. Because presepsin is mainly excreted in the urine, patients with chronic renal failure [20], especially chronic maintenance dialysis patients, tend to have high levels of presepsin. Fourth, the effect of patient background (e.g. underlying disease or the administration of chemotherapy, steroids, or antibiotics) was not analysed in detail; neither was the effect of the type, duration, or dose of antibiotic treatment or the tumour burden [21]. These parameters may have affected the levels of acute inflammatory markers. Finally, because the decision to initiate thoracentesis was based on the judgement of the pulmonary physician, a sampling bias may have been present, which could explain the very low number of cases of congestive pleural effusion representative of transudate effusion that were registered during the study period.

## Conclusions

In summary, this study showed that pleural fluid presepsin, CRP, and PCT levels may be of value as additional tools in the assessment of pleural effusions to support the differential diagnosis. High levels of pleural fluid presepsin were found in cases of empyema and parapneumonic effusion; this closely resembles the previously reported pattern of pleural fluid CRP levels. Some combinations of these pleural fluid inflammatory marker may be more likely to be clinically useful compared with these markers in isolation.

## Abbreviations

95% CI: 95% confidence interval
AUC: area under the curve
CRP: C-reactive protein
IL-6: interleukin-6
PCT: procalcitonin
ROC: receiver operator characteristics
SD: standard deviation
